# Joint Kinematics and Gait Pattern in Multiple Sclerosis: A 3D Analysis Comparative Approach

**DOI:** 10.3390/bioengineering12101067

**Published:** 2025-09-30

**Authors:** Radu Rosulescu, Mihnea Ion Marin, Elena Albu, Bogdan Cristian Albu, Marius Cristian Neamtu, Eugenia Rosulescu

**Affiliations:** 1 Department of Pathophysiology, University of Medicine and Pharmacy of Craiova, 200349 Craiova, Romania; 2 Department of Physical Therapy and Sports Medicine, University of Craiova, 200177 Craiova, Romania; rosulescu.eugenia@ucv.ro; 3 Laboratory of Innovative Techniques and Processes, Research Hub for Applied Sciences INCESA, University of Craiova, 200440 Craiova, Romania; mihnea.marin@edu.ucv.ro; 4 Department of Neurology, Faculty of Medicine, University of Medicine and Pharmacy of Craiova, 200349 Craiova, Romania; elenapinosanu@yahoo.com; 5 Emergency Clinical County Hospital Craiova, 200642 Craiova, Romania; bogdanalbu9898@gmail.com

**Keywords:** multiple sclerosis, gait, joint kinematic, 3D motion analysis

## Abstract

This cross-sectional study analyzed the lower limb (LL) behavior in terms of gait asymmetry and joints’ kinematic parameters, comparing people with multiple sclerosis (pwMS) and unaffected individuals. Methods: Data from 15 patients, EDSS ≤ 4.5, and 15 healthy control volunteers were gathered. The VICON Motion Capture System (14 infrared cameras), NEXUS software, Plug-in–Gait skeleton model and reflective markers were used to collect data for each subject during five gait cycles on a plane surface. Biomechanical analysis included evaluation of LL joints’ range of motion (ROM) bilaterally, as well as movement symmetry. Results: Comparative biomechanical analysis revealed a hierarchy of vulnerability between the groups: the ankle is the most affected joint in pwMS (*p* = 0.008–0.014), the knee is moderately affected (*p* = 0.015 in swing phase), and the hip is the least affected (*p* > 0.05 in all phases). The swing phase showed the most significant left–right asymmetry impairment, as reflected by root mean square error (RMSE) values: swing-phase RMSE = 9.306 ± 4.635 (higher and more variable) versus stance-phase RMSE = 6.363 ± 2.306 (lower and more consistent). Conclusions: MS does not affect the joints structurally; rather, it eliminates the ability to differentiate the fine-tuning control between them. The absence of significant left–right joint asymmetry differences during complete gait cycle indicates dysfunction in the global motor control.

## 1. Introduction

Motor dysfunction in multiple sclerosis (MS) can manifest in various forms, including mobility issues, muscle weakness, spasticity, and disorders affecting coordination and balance [1,2]. Around 41% of multiple sclerosis patients have gait disturbances that increase the risk of falls and impair daily life [3,4]. Gait disturbances are one of the leading causes of disability in people with multiple sclerosis (pwMS) and, from the patients’ perspective, represent the most challenging symptom [1,5]. Gait disturbances in MS can be caused by multiple neurological deficits, such as impaired motor function, coordination, or perception, and range from simple to complex etiologies [6]. Common contributing factors include balance problems, spasticity, muscle weakness, altered motor control, and gait-induced fatigue [6,7]. Moreover, as the disease progresses, gait deficits may worsen over time [8].

There are multiple methods for diagnosing gait disorders, including performance-based walking tests [9], observation of spatiotemporal gait parameters [10], dedicated gait analysis using specialized equipment [11], and physical examination [12]. Currently, three-dimensional (3D) gait analysis is the gold standard for evaluating gait abnormalities [13].

A recent study of gait patterns analysis revealed that knee flexion during the swing phase, ankle dorsiflexion at initial ground contact, and ankle plantarflexion during swing are often reduced [14,15]. Spatiotemporal gait alterations in MS have been clearly described, including reduced gait speed, decreased step length, and increased step width [16]. However, the authors emphasize that there is currently no clear consensus on the nomenclature best suited to describe common gait patterns in clinical contexts, and the heterogeneity of etiologies complicates the description of typical MS gait patterns. Therefore, the aim of the above-mentioned study was to identify common gait patterns typical of MS, describe their key characteristics, and assess potential underlying causes [14]. Using a Delphi method, the research team, in collaboration with 20 international experts, identified six key gait patterns associated with MS.

It is already known that loss of mobility in pwMS significantly diminishes quality of life, and that muscle weakness, fatigue, loss of coordination, and spasticity commonly manifest as changes in gait and balance, which ultimately lead to mobility loss [11]. Subtle changes in gait and balance are evident in pwMS even in the earliest stages of the disease and can be measured using advanced motion analysis techniques [17,18,19]. Furthermore, three out of four pwMS report gait dysfunction and restricted mobility [12,20], which are significant factors limiting quality of life [21].

Despite the well-established importance of walking function in PwMS, only a few studies have specifically monitored gait deterioration using quantitative assessments. A study reported by Paltamaa et al. [22] investigated gait disturbances in a cohort of 109 pwMS with mild disability (median EDSS score 2.0), showing a slight decrease in walking ability, according to the 6 min walk test (6MWT) results, over 2 years. In contrast, Spain et al. [23] found no changes in balance or gait in PwMS with mild impairment over 18 months using wearable sensors equipped with accelerometers and goniometers. Based on these inconclusive observations regarding gait pattern identification in MS, Zörner et al. [24] proposed tracking and characterizing gait evolution in pwMS over 4 years, using both clinical gait measures and treadmill-based gait kinematic analysis. Precise, objective tracking of gait deterioration in pwMS is uncommon, likely due to its time-consuming nature and the often-limited resources available in daily clinical practice.

In the same context of detecting gait patterns, a meta-analysis of MS gait impairment showed that most study participants had low EDSS scores, which could have reduced the study’s external validity and findings, also noting significant differences in monitoring methods and evaluation procedures [25]. General fatigability experienced by patients, which is frequently measured using the 6MWT, has been the focus of numerous gait analysis research in MS. For example, Abasıyanık et al. [26] investigated muscle strength, gait, balance, and reaction time, and their results were supported by other studies showing reduced walking speed during or after the 6MWT [27,28,29,30]. The findings suggested that following the 6MWT, there is deterioration in spatiotemporal, variability, asymmetry, regularity, stability, kinetic, and kinematic gait parameters. Moreover, most gait parameters showed greater deterioration in MS patients with moderate-to-severe disability compared to those mildly disabled.

Objective gait measurements allow for an assessment of gait quality and performance, including variability and asymmetry, providing important information to complement the neurological examination of pwMS, as they are susceptible to symptom evolution, enabling early diagnosis and evaluation of therapeutic interventions [31,32,33,34,35]. Collected data from wearable devices with inertial sensors have proven effective for objective gait evaluations [33,34,36]. The authors of that study showed that gait analysis using inertial sensors can provide objective information about the gait of pwMS. They observed that gait parameters, expressed as average values over gait cycles, achieved “excellent” intra-session reliability from three gait cycles, whereas parameters describing gait variability and asymmetry tended to reach higher Intraclass Correlation Coefficients (ICC) values when the analysis included more gait cycles. Notably, using six gait cycles, the variability and asymmetry of step length, as well as the variability and asymmetry of step velocity, demonstrated “good” reliability and should be further explored for their potential contribution to early diagnosis and monitoring of symptoms in MS.

Gait analysis by specialized equipment has enabled the identification of specific kinematic changes, displaying a decrease in step length and single-limb support time, slower gait speed, increased step width, and reduced ankle dorsiflexion angle and propulsive force [15,37,38]. However, the authors failed to define a specific gait pattern in MS, unlike in other neurological disorders such as Parkinson’s disease or stroke. Several studies described reduced gait speed and step length, reduced range of motion (ROM) in the lower limbs, and reduced dynamic stability in MS patients [18,39,40,41,42,43]. In the aforementioned studies, however, patients and healthy controls walked at self-selected speeds, resulting in significant inter-group speed differences. Because most gait parameters are strongly influenced by walking speed [44], the slower walking speed of patients compared to controls is a significant confounding factor, limiting the characterization of MS-related gait pathophysiology [10,13,45]. Although some studies have provided cross-sectional characterizations of gait disturbances in MS, longitudinal evaluations are rare despite the fact that monitoring disease progression and gait dysfunction over time is clinically important as it can help to adjust and optimize treatment strategies throughout the disease course [22,46,47,48].

Attempts have been made to identify distinct gait patterns in pwMS, with researchers trying to develop a gait model for subjects with MS and mild disability. This model was characterized by increased hip and knee flexion at initial contact, followed by reduced hip extension and reduced ankle plantarflexion during the support period compared to a nondisabled control group [17,42,49]. In the swing period, increased hip flexion was reported. Recent research on gait disorders in MS patients with mild disability using 3D gait analysis revealed that these subjects walked with a reduced speed, step length, and cadence compared to healthy individuals [50]. These alterations seem related to an increased risk of falls in these patients [50,51]. In discussing their results about other studies, the authors noted that these changes are more pronounced in MS patients with signs of spasticity [42,50]. Furthermore, at the ankle joint, several authors have described reduced ankle dorsiflexion at initial contact and reduced plantarflexion in the pre-swing phase [48]. The importance of this approach is underscored by the fact that in such individuals, these deficits were not detected clinically via standard tests [13,50,52,53].

Biomechanical gait parameters can be obtained using three-dimensional (3D) motion capture systems. To date, these systems have recorded spatiotemporal gait kinematics and kinetics (speed, cadence, and step length) in MS subjects with mild disability (e.g., a score ≤3 on the Expanded Disability Status Scale (EDSS) [54]. However, they have not focused on temporal parameters. Temporal parameters correspond to temporal events (expressed as a percentage of the gait cycle) and related kinematic and kinetic measures.

Therefore, the aim of our study was to perform a comparative analysis of lower limb behavior during gait by the VICON motion capture system, focusing on asymmetry and kinematic parameters, between people with multiple sclerosis (pwMS) with an EDSS ≤ 4.5 and healthy controls.

## 2. Materials and Methods

### 2.1. Participants

This comparative, observational cross-sectional-type study included 15 voluntary patients (8 females and 7 males), with a disease duration mean of 8.93 ± 3.75 years (min 4, max 16), mean EDSS 2.06 ± 1.15 (min 1, max 4.5), a mean age of 49.8 ± 4.55 years, a mean weight of 77.6 ± 12.51 kg, and a mean body mass index (BMI) of 26.1 ± 3.19. This study also included a control group of 15 healthy subjects (8 females and 7 males, mean age 49 ± 3.87 years; mean weight 75 ± 11.41 kg; mean BMI 26 ± 3.26) with no associated locomotor pathology. We did not make an assessment according to gender distribution, and there was no bias from this point of view. All MS patients in this study were treated with disease-modifying therapies (DMTs: 5 patients with glatiramer acetate, 10 patients with interferon beta-1b).

Patients from a local hospital were selected based on inclusion and exclusion criteria. Inclusion criteria: confirmed diagnosis of relapsing-remitting MS based on the 2017 McDonald criteria [55]; age 18–64 years; no relapse in the last 30 days and no monthly use of medications for ongoing relapse (e.g., corticosteroids); ability to walk with or without an assistive device; and EDSS ≤ 4.5 (note: a score > 4.5 indicates that constant bilateral assistance is required to walk approximately 20 m) [54]. Exclusion criteria: any musculoskeletal and/or neurological disorders that could affect gait and balance (other than MS); EDSS score > 4.5; secondary progressive MS; confinement to a wheelchair or bed; age > 64 years; severe communication difficulties; severe muscle weakness and spasticity; or relapse in the last 3 months.

Healthy control volunteers were freely recruited and chosen based on their anthropometric characteristics, which were comparable to those of MS patients.

Written informed consent was given by all participants, and this study complied with the 2013 version of the Declaration of Helsinki’s ethical guidelines for biomedical research involving human subjects. This study received approval from the local Research Ethics and Deontology Commission of the University of Medicine and Pharmacy Craiova (approval No. 104/2025).

### 2.2. Experimental Protocol and Data Collection

This research was carried out between January and July 2025. A thorough assessment of individuals with relapsing-remitting multiple sclerosis was part of it. These subjects underwent a clinical–functional assessment using the EDSS score, as well as a qualitative gait evaluation in which we observed the progression of gait phases (heel strike, single support, double support, swing phase) and the kinematic behavior of the lower limb as a kinetic chain at each joint.

**Figure 1:**
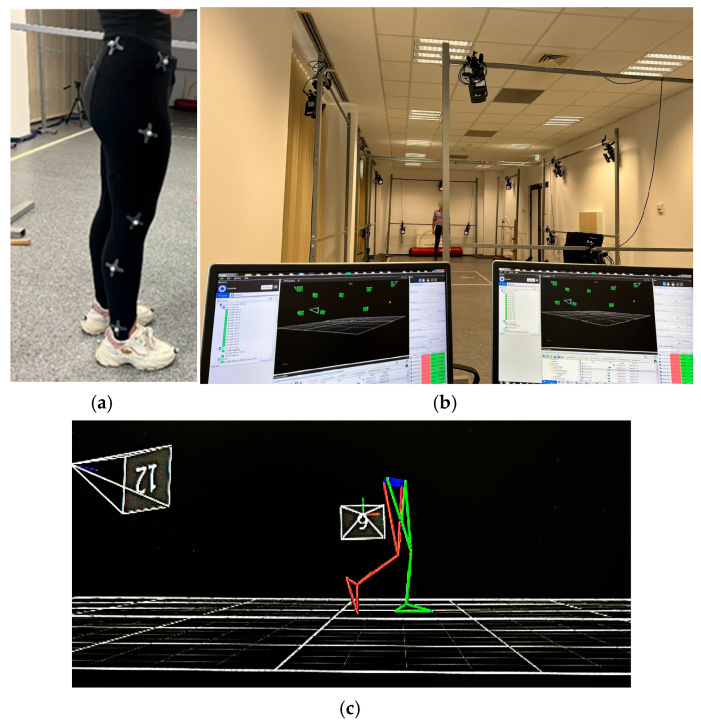
Vicon motion analysis system: (**a**) the right-side view of marker set for gait analysis; (**b**) Vicon Motion Capture System setup at the INCESA Laboratory; (**c**) gait capture example.

Quantitative gait analysis involved a biomechanical analysis of the lower limbs using the 14-camera Vicon motion analysis system (Oxford Metrics Ltd., Oxford, UK), NEXUS software version V2.10, Plug-in–Gait—lower limb skeleton model and 16 reflective markers, 14 mm diameter, available at the Laboratory of Innovative Techniques and Processes, INCESA, of University of Craiova (www.incesa.ro). Lower limb markers were placed on the anterior and posterior superior iliac spines, lateral middle third of the thigh, lateral knee, lateral middle third of the calf, external malleolus, second metatarsal head, and heel. Camera calibration was performed prior to data collection, set up at 100 Hz. The assessment was performed during five gait cycles to analyze the lower limb (LL) during gait on a plane surface, following the trajectory of the LL’s main important points (hip, knee, malleolus), and variation in angular amplitude for hip, knee and ankle joints of both lower limbs (Figure 1).

The biomechanical analysis included evaluation of the range of motion (ROM) of the lower limb joints bilaterally, as well as the left–right symmetry index. A descriptive statistical analysis was performed on these data and the following values were calculated: mean, standard deviation, CV (coefficient of variation), ROM (range of motion) and SI (symmetry index).

ROM represents the total range of motion of a joint during the gait cycle, measured in degrees. ROM = Maximum Angular Value—Minimum Angular Value.

The NEXUS V2.10 database allowed for the calculation of the RMSE (root mean square error) parameter. The RMSE parameter shows how much the values differ between the left and right limbs for a study participant. The lower the RMSE, the more symmetrical the movement.

**Figure 2:**
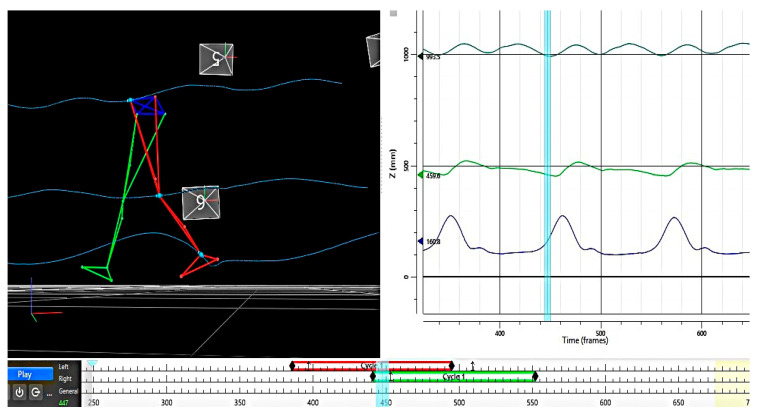
The joints’ trajectories: hip, knee, ankle.

The curves of the joints’ trajectories during the gait cycle are presented in Figure 2.

### 2.3. Data Analysis

For all study participants (30 total, 15 in each group), with the VICON complex image acquisition and analysis system, the angles of the two lower limbs’ 3 joints were measured. These measurements were collected during a gait cycle and normalized to 100 values (frames). In total, 6 joints × 100 frames × 30 subjects = 18,000 data points were collected.

The results were processed using XLStat software (an Excel add-in, https://www.xlstat.com), computing descriptive statistics for each analyzed parameter: mean, standard deviation (SD), coefficient of variation (CV), ROM, and the RMSE (root mean square error). Additionally, we performed the following statistical analyses: a *t*-test, to compare joint ROM between the two groups (ROM_MS, n = 15 vs. ROM_Control, n = 15); and calculation of the root mean square error (RMSE). A one-way ANOVA and Cohen’s effect size was performed to assess significant differences between MS patients and the control group.

RMSE was calculated, both for the entire gait cycle (100%), and separately for the stance phase (60%) and the swing phase (40%). The RMSE measures the instantaneous asymmetry between the left and right limbs throughout the entire gait cycle.

RMSE = √(Σ(Left_Value_i − Right_Value_i)^2^/n)

where:

- i = the point in the cycle (1 to 100);
- n = the total number of points (100).

These values are shown in Tables 2 and 3 for each participant of both groups.

## 3. Results

### 3.1. Comparative Analysis of ROM for the Hip, Knee, and Ankle Joints (Bilateral)

The values from the descriptive analysis and their significance are presented in Table 1. A significance *t*-test (Student’s *t*-test, α ≤ 0.05) was applied to the ROM data for each joint comparing the two groups (MS patients and controls).

Analysis of the data reveals significant ROM differences at the hip and knee joints, with more pronounced deficits in the left limb. The magnitude of the ROM deficit in the MS group relative, or versus (vs.), to controls was as follows: right hip: –21.2% (mean 37.05° vs. 29.20°); left hip: –14.8% (32.67° vs. 27.84°); right knee: –22.8% (72.79° vs. 56.20°); left knee: –16.0% (61.06° vs. 51.27°); ankle: –18.1%/–0.9% (right/left; differences not significant). These deficits are substantial, ranging from 15% to 23%.

### 3.2. Root Mean Square Error (RMSE)

The study database and the performed measurements allowed for the statistic calculation of the RMSE (root mean square error), which indicates the extent to which the movement amplitudes differ between a person’s left and right lower limbs. The smaller the RMSE value, the more symmetric the movement. Using Excel, we computed the RMSE for the whole gait cycle (100%), as well as separately for the stance phase (60% of the gait cycle) and swing phase (40% of the cycle). These values are shown in Table 2 and Table 3 for each participant of both groups.

Statistical analysis was further extended with separate one-way ANOVA tests on the RMSE values for each of the three joints inside each group (MS patients and healthy controls). The results are displayed in Table 4, corresponding to the three gait phases considered.

The one-way ANOVA test reveals that there are no significant differences in the RMSE statistical indicator in both groups MS patients, and therefore implicitly in the asymmetry at the level of the three evaluated joints (F = 0.172, *p* = 0.682), nor when comparing the left to the right side. According to the ANOVA results, in healthy subjects, the RMSE does not differ significantly, thus applying to asymmetry as well, at any of the three joints when comparing the left and right sides. In the control group, no left–right asymmetry was observed in the lower limbs for any joint or gait phase, mirroring the findings in the MS group.

The result regarding RMSE, as an indicator of bilateral joint symmetry, shows that both groups (MS and Control) present the same pattern and there are no differences between the joints in any of the gait phases. A Student’s *t*-test was then applied to analyze differences between the two groups at the global gait level; the results are presented in Table 5.

Regarding Cohen’s test, we observe that there are medium- and large-size effects for the ankle in all gait phases. The presented statistical analysis shows the existence of specific phase-dependent patterns in the MS group, bringing important information for clinicians regarding the prediction of gait disturbance progression and potential therapeutic interventions. Thus, we observed the existence of specific phase patterns, developed in subjects with MS, compared to the control group, as follows:

- Throughout the whole gait cycle, only the ankle joint is significantly affected (*p* = 0.014), whereas the hip and knee do not differ significantly (*p* > 0.05).
- During the stance phase, no joints are noticeably affected.
- Swing phase: the knee and ankle joints are significantly impacted (*p* = 0.015 and *p* = 0.008, respectively), whereas the hip remains unaffected.

The two statistical approaches (one-way ANOVA and *t*-test) are complementary. They indicate that, although bilateral joint symmetry is maintained in both groups (as per the ANOVA results), there are significant between-group differences at the knee and ankle joints when analyzing specific gait phases.

## 4. Discussion

In this observational study, we found that MS functionally affects all the lower limb joints uniformly (i.e., no particular joint is spared), and this uniform impact is a significant one. A hierarchy of vulnerability between the groups can thus be determined: the ankle is the most affected joint in MS (*p* = 0.008–0.014), the knee is moderately affected (*p* = 0.015 in swing phase), and the hip is the least affected (*p* > 0.05 in all phases). In this context, the pattern of impairment can be described as proximal–distal (impact increasing from proximal to distal joints).

As a consequence, the obtained results have several clinical implications. The absence of significant left–right joint asymmetry differences during a complete gait cycle indicates a dysfunction in global motor control rather than in individual joints, meaning that people with MS exhibit impaired postural control. Moreover, from a clinical standpoint, the swing phase shows the most significant left–right asymmetry impairment, as reflected by the ankle RMSE values: swing-phase RMSE = 9.21 ± 5.51 (higher and more variable) versus stance-phase RMSE = 6.18 ± 3.15 (lower and more consistent).

Comparing our findings with the literature, we found that regarding kinematic parameters, Severini et al. [43] observed an increase in pelvic tilt and displacement throughout the gait cycle. At the hip joint, studies have described a decrease in maximal extension during stance in patients with mild-to-moderate impairment, while in patients with lesser involvement (EDSS < 4), no significant hip differences were found [56]. For the knee joint, prior studies reported a decrease in maximum swing-phase flexion [39,43], whereas at the ankle joint, several studies observed reduced dorsiflexion during the stance phase [39,41,43]. These findings support our observations as they indicate a predominantly ankle-level impairment in MS.

According to Filli et al. [39], MS patients show a bilateral decrease in step length, which is linked to a loss in lower limb joint range of motion. While hip ROM was preserved, significant reductions in ROM were noted, especially at the knee and at the ankle. The more pronounced ROM restriction at the knee and ankle led to substantial left–right asymmetry, whereas inter-limb coordination parameters and double-support duration showed only minor deviations in patients. These results confirm our findings, as supported by the RMSE indicator, which assessed left–right inter-joint symmetry in the context of a global asymmetry relative to healthy controls. Similarly, Zörner et al. [24] showed that over a 4-year follow-up of MS patients, those with more pronounced deterioration of gait function had greater reductions in ankle and knee ROM over time. Our results further highlight how crucial monitoring patients’ gait function using objective, quantitative measurements is. Fritz et al. [47] have observed that gait analysis, when combined with clinical, electrophysiological, and imaging evaluations, can improve the functional prediction, especially regarding progression of gait abnormalities.

Integrating information from kinematic and kinetic measurements allows for a thorough gait assessment in people with progressive MS; however, the kinematic changes in this patient category affect multiple joints, and the sheer volume of data presents challenges that limit clinical interpretation. Massot et al. [57] emphasized, as we also observed, that detecting subclinical gait and balance deficits requires the use of 3D motion capture systems [58]. Since gait initiation demands coordinated joint performance in both posture and locomotion, using three-dimensional gait analysis allows for the revelation of gait and balance problems in MS [59].

From a clinical perspective, our results complement those of other researchers. We observed that healthy subjects maintain subtle flexibility in inter-articular control (especially during the swing phase). In contrast, MS patients lose this flexibility, developing a uniform and rigid gait pattern. Therefore, the ability to predict when and how walking function may deteriorate in people with MS could enable targeted interventions to preserve ambulatory function, quality of life, and independence.

Characterizing gait in MS could serve purposes similar to those in other conditions. Understanding the factors that contribute to MS gait impairments and correctly identifying these factors through detailed analysis can help standardize and better comprehend suitable treatment alternatives.

One of this study’s limitations could be the MS patients’ medication (DMTs: 5 patients received glatiramer acetate, 10 patients received interferon beta-1b), but this did not significantly influence the study results, because as far as is known, fampridine is the only medical drug used to alleviate gait function in MS [60]. The main limitation of this study is the small number of subjects in both groups, 15 pwMS and 15 healthy control subjects; also, there may be potential data collection errors due to subjects’ difficulty in fully understanding the requirement to walk naturally.

## 5. Conclusions

In this study, MS patients showed a non-selective joint involvement. According to this analysis, MS does not affect individual joints at a structural level; rather, it eliminates the ability to differentiate the fine-tuning control between them. The absence of significant left–right joint asymmetry differences during a complete gait cycle indicates a dysfunction in global motor control and an impaired postural control in MS patients. The swing phase is more affected, making the motor control during this phase more difficult for pwMS, although the degree of impairment remains equal in all joints. Therefore, there is a need for comprehensive rehabilitation and, because MS does not target any one joint preferentially, neurorehabilitation strategies must address all joints, and clinical gait evaluation should include all segments.

## Figures and Tables

**Table 1:** Hip, knee and ankle joint range of motion (ROM) and Student’s *t*-test results (ROM comparison between groups).

Joint	Side	Control (Mean ± SD)	MS (Mean ± SD)	Control Minimum/ Maximum	MS Minimum/ Maximum	Difference	*p*-Value
Hip	Right	37.05 ± 10.13	29.20 ± 9.33	28.39/59.92	18.95/44.09	−7.85°	0.034 *
	Left	32.67 ± 3.06	27.84 ± 6.69	26.64/39.01	22.97/39.01	−4.83°	0.013 *
Knee	Right	72.79 ± 29.57	56.20 ± 7.33	50.81/139.41	47.74/66.70	−16.59°	0.048 *
	Left	61.06 ± 5.55	51.27 ± 10.87	50.36/67.303	39.02/67.98	−9.79°	0.010 *
Ankle	Right	34.59 ± 22.35	28.33 ± 4.58	23.64/111.95	24.04/36.95	−6.26°	0.265
	Left	27.39 ± 4.17	27.14 ± 1.52	20.53/36.37	24.23/28.63	−0.25°	0.851

* *p*-values < 0.05 are considered significant.

**Table 2:** RMSE for healthy individuals (control group).

Control Subject No.	RMSE—Hip	RMSE—Knee	RMSE—Foot
S1	2.09	2.92	5.93
S2	4.09	14.53	7.15
S3	3.50	10.14	3.53
S4	4.32	5.84	4.58
S5	3.07	8.41	4.24
S6	4.48	16.30	12.06
S7	1.36	3.69	3.29
S8	0.70	3.31	2.60
S9	1.37	5.29	3.45
S10	0.91	2.68	3.84
S11	0.82	2.58	4.21
S12	3.70	5.00	3.32
S13	1.76	2.59	2.35
S14	2.34	3.76	6.53
S15	2.04	3.15	7.18
1st Quartile	1.36	3.03	3.38
Median	2.09	3.76	4.21
3rd Quartile	3.60	7.13	6.23
Mean	2.44	6.01	4.95
Standard deviation	1.32	4.41	2.51
CV	54.38	73.41	50.70

**Table 3:** RMSE for MS patients.

MS Patient No.	RMSE—Hip	RMSE—Knee	RMSE—Foot
P1	4.23	9.11	15.44
P2	3.45	6.12	5.25
P3	1.60	6.12	5.71
P4	1.60	10.83	5.71
P5	4.07	7.91	5.51
P6	4.21	9.85	16.44
P7	3.94	7.12	6.25
P8	1.96	5.12	6.71
P9	2.60	10.02	5.67
P10	4.97	8.58	6.51
P11	4.06	8.11	14.44
P12	2.45	7.12	4.25
P13	0.60	5.12	4.71
P14	1.99	11.83	5.99
P15	3.07	9.11	4.51
1st Quartile	1.98	6.62	5.38
Median	3.07	7.91	5.71
3rd Quartile	4.07	9.48	6.61
Mean	2.99	8.04	7.54
Standard deviation	1.27	2.02	4.16

**Table 4:** ANOVA results for the RMSE in the control group and the MS group.

Group	RMSE Metric	F	*p*	Conclusion
Control	RMSE for the entire gait cycle	0.695	0.411	No significant differences
	RMSE for stance phase	0.060	0.808	No significant differences
	RMSE for swing phase	1.32	0.260	No significant differences
MS	RMSE for the entire gait cycle	0.172	0.682	No significant differences
	RMSE for stance phase	0.190	0.666	No significant differences
	RMSE for swing phase	0.012	0.915	No significant differences

**Table 5:** Comparative results of MS vs. control groups for overall gait phases and for each joint.

Phase	Parameter		MS Group	Control Group	Difference/ Cohen’s d’ Test	*p*-Value	Interpretation/Significance
Complete cycle	Mean ± SD	Hip	2.98 ± 1.26	2.44 ± 1.31	+0.54/0.58	0.296	Not significant
		Knee	8.04 ± 2.02	6.02 ± 4.41	+2.02/0.65	0.130	Not significant
		Ankle	7.54 ± 4.16	4.93 ± 2.52	+2.61/1.46	0.014 *	Significant
	F-value		0.172	0.695			Not significant
	*p*-value		0.682	0.411			Not significant
	R^2^		0.006	0.024			Not significant
Stance phase	Mean ± SD	Hip	2.60 ± 1.61	2.08 ± 1.52	+0.52/0.48	0.227	Not significant
		Knee	6.55 ± 1.00	5.28 ± 5.90	+1.27/0.3	0.400	Not significant
		Ankle	6.18 ± 3.15	4.86 ± 3.26	+1.32/0.57	0.170	Not significant
	F-value		0.190	0.060			Not significant
	*p*-value		0.666	0.808			Not significant
	R^2^		0.007	0.002			Not significant
Swing phase	Mean ± SD	Hip	3.21 ± 0.90	2.60 ± 1.64	+0.61/0.53	0.237	Not significant
		Knee	9.40 ± 3.76	5.81 ± 3.53	+3.59/1.44	0.015 *	Significant
		Ankle	9.21 ± 5.51	4.59 ± 2.11	+4.62/3.10	0.008 *	Significant
	F-value		0.012	1.320			Not significant
	*p*-value		0.915	0.260			Not significant
	R^2^		0.000	0.045			Not significant

* *p*-values < 0.05 are considered significant.

## Data Availability

The data are available in the tables in this manuscript. Other data used to support the findings of this study are available from the corresponding author upon request.

## References

[1] Huang W.-J., Chen W.-W., Zhang X. (2017). Multiple Sclerosis: Pathology, Diagnosis and Treatments. Exp. Ther. Med..

[2] Schapiro R.T. (1994). Symptom Management in Multiple Sclerosis. Ann. Neurol..

[3] Finlayson M.L., Peterson E.W., Cho C.C. (2006). Risk Factors for Falling Among People Aged 45 to 90 Years with Multiple Sclerosis. Arch. Phys. Med. Rehabil..

[4] Slavkovic S., Golubovic S., Vojnovic M., Nadj C. (2019). Influence of Cognitive and Motor Abilities on the Level of Current Functioning in People with Multiple Sclerosis. Slov. J. Public Health.

[5] Yozbatıran N., Baskurt F., Baskurt Z., Ozakbas S., Idiman E. (2006). Motor Assessment of Upper Extremity Function and Its Relation with Fatigue, Cognitive Function and Quality of Life in Multiple Sclerosis Patients. J. Neurol. Sci..

[6] Bethoux F. (2013). Gait Disorders in Multiple Sclerosis. Continuum.

[7] Eken M.M., Richards R., Beckerman H., Van Der Krogt M., Gerrits K., Rietberg M., De Groot V., Heine M. (2020). Quantifying Muscle Fatigue during Walking in People with Multiple Sclerosis. Clin. Biomech..

[8] Galea M.P., Cofré Lizama L.E., Butzkueven H., Kilpatrick T.J. (2017). Gait and Balance Deterioration over a 12-Month Period in Multiple Sclerosis Patients with EDSS Scores ≤ 3.0. NeuroRehabilitation.

[9] Bennett S.E., Bromley L.E., Fisher N.M., Tomita M.R., Niewczyk P. (2017). Validity and Reliability of Four Clinical Gait Measures in Patients with Multiple Sclerosis. Int. J. MS Care.

[10] Lizrova Preiningerova J., Novotna K., Rusz J., Sucha L., Ruzicka E., Havrdova E. (2015). Spatial and Temporal Characteristics of Gait as Outcome Measures in Multiple Sclerosis (EDSS 0 to 6.5). J. Neuroeng. Rehabil..

[11] Shanahan C.J., Boonstra F.M.C., Cofré Lizama L.E., Strik M., Moffat B.A., Khan F., Kilpatrick T.J., Van Der Walt A., Galea M.P., Kolbe S.C. (2018). Technologies for Advanced Gait and Balance Assessments in People with Multiple Sclerosis. Front. Neurol..

[12] Hobart J.C., Riazi A., Lamping D.L., Fitzpatrick R., Thompson A.J. (2003). Measuring the Impact of MS on Walking Ability: The 12-Item MS Walking Scale (MSWS-12). Neurology.

[13] Sosnoff J.J., Sandroff B.M., Motl R.W. (2012). Quantifying Gait Abnormalities in Persons with Multiple Sclerosis with Minimal Disability. Gait Posture.

[14] Timmermans S.T., Van Der Krogt M.M., Rietberg M.B., Beckerman H., De Groot V. (2025). A Delphi Study to Identify Key Gait Patterns and Their Potential Causes in People with Multiple Sclerosis. J. Rehabilitation Med..

[15] Coca-Tapia M., Cuesta-Gómez A., Molina-Rueda F., Carratalá-Tejada M. (2021). Gait Pattern in People with Multiple Sclerosis: A Systematic Review. Diagnostics.

[16] Comber L., Galvin R., Coote S. (2017). Gait Deficits in People with Multiple Sclerosis: A Systematic Review and Meta-Analysis. Gait Posture.

[17] Benedetti M.G., Piperno R., Simoncini L., Bonato P., Tonini A., Giannini S. (1999). Gait Abnormalities in Minimally Impaired Multiple Sclerosis Patients. Mult. Scler..

[18] Martin C.L., Phillips B.A., Kilpatrick T.J., Butzkueven H., Tubridy N., McDonald E., Galea M.P. (2006). Gait and Balance Impairment in Early Multiple Sclerosis in the Absence of Clinical Disability. Mult. Scler..

[19] Kalron A., Achiron A., Dvir Z. (2011). Muscular and Gait Abnormalities in Persons with Early Onset Multiple Sclerosis. J. Neurol. Phys. Ther..

[20] Kister I., Chamot E., Salter A.R., Cutter G.R., Bacon T.E., Herbert J. (2013). Disability in Multiple Sclerosis: A Reference for Patients and Clinicians. Neurology.

[21] Créange A., Serre I., Levasseur M., Audry D., Nineb A., Boërio D., Moreau T., Maison P., Sindefi-Sep R. (2007). Walking Capacities in Multiple Sclerosis Measured by Global Positioning System Odometer. Mult. Scler..

[22] Paltamaa J., Sarasoja T., Leskinen E., Wikström J., Mälkiä E. (2008). Measuring Deterioration in International Classification of Functioning Domains of People with Multiple Sclerosis Who Are Ambulatory. Phys. Ther..

[23] Spain R.I., Mancini M., Horak F.B., Bourdette D. (2014). Body-Worn Sensors Capture Variability, but Not Decline, of Gait and Balance Measures in Multiple Sclerosis over 18 Months. Gait Posture.

[24] Zörner B., Hostettler P., Meyer C., Killeen T., Gut P., Linnebank M., Weller M., Straumann D., Filli L. (2022). Prognosis of Walking Function in Multiple Sclerosis Supported by Gait Pattern Analysis. Mult. Scler. Relat. Disord..

[25] Vienne-Jumeau A., Quijoux F., Vidal P.P., Ricard D. (2019). Value of Gait Analysis for Measuring Disease Severity Using Inertial Sensors in Patients with Multiple Sclerosis: Protocol for a Systematic Review and Meta-Analysis. Syst. Rev..

[26] Abasıyanık Z., Kahraman T., Veldkamp R., Ertekin Ö., Kalron A., Feys P. (2022). Changes in Gait Characteristics During and Immediately After the 6-Minute Walk Test in Persons with Multiple Sclerosis: A Systematic Review. Phys. Ther..

[27] Goldman M.D., Marrie R.A., Cohen J.A. (2008). Evaluation of the Six-Minute Walk in Multiple Sclerosis Subjects and Healthy Controls. Mult. Scler..

[28] McLoughlin J.V., Barr C.J., Patritti B., Crotty M., Lord S.R., Sturnieks D.L. (2016). Fatigue Induced Changes to Kinematic and Kinetic Gait Parameters Following Six Minutes of Walking in People with Multiple Sclerosis. Disabil. Rehabil..

[29] Ramari C., Moraes A.G., Tauil C.B., Von Glehn F., Motl R., De David A.C. (2018). Knee Flexor Strength and Balance Control Impairment May Explain Declines during Prolonged Walking in Women with Mild Multiple Sclerosis. Mult. Scler. Relat. Disord..

[30] Drebinger D., Rasche L., Kroneberg D., Althoff P., Bellmann-Strobl J., Weygandt M., Paul F., Brandt A.U., Schmitz-Hübsch T. (2020). Association Between Fatigue and Motor Exertion in Patients with Multiple Sclerosis—A Prospective Study. Front. Neurol..

[31] Motti Ader L.G., Greene B.R., McManus K., Tubridy N., Caulfield B. (2020). Short Bouts of Gait Data and Body-Worn Inertial Sensors Can Provide Reliable Measures of Spatiotemporal Gait Parameters from Bilateral Gait Data for Persons with Multiple Sclerosis. Biosensors.

[32] Lord S., Galna B., Rochester L. (2013). Moving Forward on Gait Measurement: Toward a More Refined Approach. Mov. Disord..

[33] Vienne A., Barrois R.P., Buffat S., Ricard D., Vidal P.-P. (2017). Inertial Sensors to Assess Gait Quality in Patients with Neurological Disorders: A Systematic Review of Technical and Analytical Challenges. Front. Psychol..

[34] Frechette M.L., Meyer B.M., Tulipani L.J., Gurchiek R.D., McGinnis R.S., Sosnoff J.J. (2019). Next Steps in Wearable Technology and Community Ambulation in Multiple Sclerosis. Curr. Neurol. Neurosci. Rep..

[35] Hubble R.P., Naughton G.A., Silburn P.A., Cole M.H. (2015). Wearable Sensor Use for Assessing Standing Balance and Walking Stability in People with Parkinson’s Disease: A Systematic Review. PLoS ONE.

[36] Simon S.R. (2004). Quantification of Human Motion: Gait Analysis—Benefits and Limitations to Its Application to Clinical Problems. J. Biomech..

[37] Gor-García-Fogeda M.D., Cano De La Cuerda R., Carratalá Tejada M., Alguacil-Diego I.M., Molina-Rueda F. (2016). Observational Gait Assessments in People with Neurological Disorders: A Systematic Review. Arch. Phys. Med. Rehabil..

[38] Crenshaw S.J., Royer T.D., Richards J.G., Hudson D.J. (2006). Gait Variability in People with Multiple Sclerosis. Mult. Scler..

[39] Filli L., Sutter T., Easthope C.S., Killeen T., Meyer C., Reuter K., Lörincz L., Bolliger M., Weller M., Curt A. (2018). Profiling Walking Dysfunction in Multiple Sclerosis: Characterisation, Classification and Progression over Time. Sci. Rep..

[40] Givon U., Zeilig G., Achiron A. (2009). Gait Analysis in Multiple Sclerosis: Characterization of Temporal–Spatial Parameters Using GAITRite Functional Ambulation System. Gait Posture.

[41] Socie M.J., Motl R.W., Pula J.H., Sandroff B.M., Sosnoff J.J. (2013). Gait Variability and Disability in Multiple Sclerosis. Gait Posture.

[42] Pau M., Coghe G., Corona F., Marrosu M.G., Cocco E. (2015). Effect of Spasticity on Kinematics of Gait and Muscular Activation in People with Multiple Sclerosis. J. Neurol. Sci..

[43] Severini G., Manca M., Ferraresi G., Caniatti L.M., Cosma M., Baldasso F., Straudi S., Morelli M., Basaglia N. (2017). Evaluation of Clinical Gait Analysis Parameters in Patients Affected by Multiple Sclerosis: Analysis of Kinematics. Clin. Biomech..

[44] Broom L., Ellison B.A., Worley A., Wagenaar L., Sörberg E., Ashton C., Bennett D.A., Buchman A.S., Saper C.B., Shih L.C. (2017). A Translational Approach to Capture Gait Signatures of Neurological Disorders in Mice and Humans. Sci. Rep..

[45] Van Der Linden M.L., Scott S.M., Hooper J.E., Cowan P., Mercer T.H. (2014). Gait Kinematics of People with Multiple Sclerosis and the Acute Application of Functional Electrical Stimulation. Gait Posture.

[46] Cutter G.R. (1999). Development of a Multiple Sclerosis Functional Composite as a Clinical Trial Outcome Measure. Brain.

[47] Fritz N.E., Newsome S.D., Eloyan A., Marasigan R.E.R., Calabresi P.A., Zackowski K.M. (2015). Longitudinal Relationships among Posturography and Gait Measures in Multiple Sclerosis. Neurology.

[48] Cadavid D., Jurgensen S., Lee S. (2013). Impact of Natalizumab on Ambulatory Improvement in Secondary Progressive and Disabled Relapsing-Remitting Multiple Sclerosis. PLoS ONE.

[49] Huisinga J.M., Schmid K.K., Filipi M.L., Stergiou N. (2013). Gait Mechanics Are Different Between Healthy Controls and Patients with Multiple Sclerosis. J. Appl. Biomech..

[50] Molina-Rueda F., Fernández-Vázquez D., Navarro-López V., Miangolarra-Page J.C., Carratalá-Tejada M. (2022). The Timing of Kinematic and Kinetic Parameters during Gait Cycle as a Marker of Early Gait Deterioration in Multiple Sclerosis Subjects with Mild Disability. J. Clin. Med..

[51] Kasser S.L., Jacobs J.V., Foley J.T., Cardinal B.J., Maddalozzo G.F. (2011). A Prospective Evaluation of Balance, Gait, and Strength to Predict Falling in Women with Multiple Sclerosis. Arch. Phys. Med. Rehabil..

[52] Bethoux F., Bennett S. (2011). Evaluating Walking in Patients with Multiple Sclerosis. Int. J. MS Care.

[53] Fernández-Vázquez D., Calvo-Malón G., Molina-Rueda F., López-González R., Carratalá-Tejada M., Navarro-López V., Miangolarra-Page J.C. (2023). Kinematic Gait Analysis in People with Mild-Disability Multiple Sclerosis Using Statistical Parametric Mapping: A Cross-Sectional Study. Sensors.

[54] Kurtzke J.F. (1983). Rating Neurologic Impairment in Multiple Sclerosis: An Expanded Disability Status Scale (EDSS). Neurology.

[55] Thompson A.J., Banwell B.L., Barkhof F., Carroll W.M., Coetzee T., Comi G., Correale J., Fazekas F., Filippi M., Freedman M.S. (2018). Diagnosis of Multiple Sclerosis: 2017 Revisions of the McDonald Criteria. Lancet Neurol..

[56] Kalron A., Dvir Z., Givon U., Baransi H., Achiron A. (2014). Gait and Jogging Parameters in People with Minimally Impaired Multiple Sclerosis. Gait Posture.

[57] Massot C., Bègue J., Simoneau-Buessinger E., Donze C., Caderby T., Leteneur S. (2025). Patients with Multiple Sclerosis and Low Disability Display Cautious Rotational Behavior during Gait Initiation. Clin. Biomech..

[58] Cofré Lizama L.E., Khan F., Lee P.V., Galea M.P. (2016). The Use of Laboratory Gait Analysis for Understanding Gait Deterioration in People with Multiple Sclerosis. Mult. Scler..

[59] Massot C., Simoneau-Buessinger E., Agnani O., Donze C., Leteneur S. (2019). Anticipatory Postural Adjustment during Gait Initiation in Multiple Sclerosis Patients: A Systematic Review. Gait Posture.

[60] Ahdab R., Shatila M.M., Shatila A.R., Khazen G., Freiha J., Salem M., Makhoul K., El Nawar R., El Nemr S., Ayache S.S. (2019). Cortical Excitability Measures May Predict Clinical Response to Fampridine in Patients with Multiple Sclerosis and Gait Impairment. Brain Sci..

